# Supplementary material for: Evaluation of the horizontal approach to the medial malleolar facet in sagittal talar fractures through dorsiflexion and plantarflexion positions
Source: PLoS One. 2024 May 15;19(5):e0295350. doi: 10.1371/journal.pone.0295350 (PMC11095721; doi:10.1371/journal.pone.0295350)
Supplement: S1 Data — (DOCX) [file pone.0295350.s001.docx]

**Minimum data set**

| Gender | Age | TA（mm^2^） | FAA（mm^2^） | FAP（mm^2^） | DA（mm^2^） | (DA-FA)/TA（%） | PA（mm^2^） | (PA-FAA)/TA（%） | UA（mm^2^） | L1（mm） | L2（mm） | L3（mm） |
| --- | --- | --- | --- | --- | --- | --- | --- | --- | --- | --- | --- | --- |
| Male | 18 | 489.81 | 123.76 | 146.52 | 309.28 | 33.23 | 203.27 | 16.23 | 25.23 | 11.34 | 5.37 | 4.05 |
| Male | 43 | 679.33 | 181.72 | 206.44 | 454.93 | 36.58 | 276.21 | 13.91 | 32.29 | 11.84 | 5.39 | 5.85 |
| Male | 25 | 692.06 | 187.56 | 219.69 | 463.68 | 35.26 | 282.98 | 13.79 | 36.36 | 13.24 | 6.22 | 5.67 |
| Male | 54 | 645.02 | 174.25 | 198.07 | 432.16 | 36.29 | 279.07 | 16.25 | 27.73 | 12.96 | 7.09 | 3.95 |
| Male | 24 | 523.74 | 136.97 | 174.58 | 350.91 | 33.67 | 204.58 | 12.91 | 23.11 | 11.38 | 6.08 | 4.99 |
| Male | 46 | 536.82 | 142.69 | 179.61 | 364.24 | 34.39 | 216.37 | 13.73 | 23.68 | 10.35 | 7.32 | 4.68 |
| Male | 54 | 562.89 | 156.84 | 183.53 | 371.1 | 33.32 | 223.39 | 11.82 | 25.6 | 10.89 | 4.98 | 5.04 |
| Male | 54 | 628.63 | 164.58 | 171.85 | 418.2 | 39.19 | 244.87 | 12.77 | 31.37 | 9.23 | 6.45 | 5.21 |
| Male | 41 | 594.84 | 150.31 | 162.35 | 404.51 | 40.71 | 288.65 | 23.26 | 30.51 | 12.03 | 7.02 | 4.81 |
| Female | 48 | 449.16 | 115.31 | 139.72 | 300.94 | 35.89 | 179.72 | 14.34 | 21.71 | 9.79 | 4.33 | 3.68 |
| Female | 30 | 488.78 | 119.16 | 142.93 | 317.48 | 35.71 | 182.93 | 13.05 | 15.92 | 10.54 | 4.53 | 5.09 |
| Female | 45 | 560.58 | 145.9 | 176.86 | 365.59 | 33.67 | 216.86 | 12.66 | 17.47 | 8.69 | 5.88 | 4.06 |
| Female | 52 | 463.33 | 121.25 | 150.22 | 336.68 | 40.24 | 198.58 | 16.7 | 16.32 | 10.08 | 5.69 | 4.87 |
| Female | 21 | 501.32 | 135.86 | 164.82 | 351.28 | 37.19 | 205.84 | 13.96 | 19.52 | 10.58 | 6.01 | 3.95 |
| Female | 19 | 571.44 | 152.35 | 179.95 | 359.84 | 31.48 | 215.52 | 11.05 | 18.64 | 9.06 | 6.38 | 4.61 |
| Female | 36 | 513.39 | 131.69 | 168.59 | 349.87 | 35.31 | 208.96 | 15.05 | 17.65 | 12.01 | 4.96 | 3.84 |
| Female | 37 | 425.84 | 109.83 | 139.56 | 321.25 | 42.67 | 186.25 | 17.95 | 19.63 | 10.26 | 4.25 | 4.01 |
| Female | 51 | 476.38 | 115.84 | 151.23 | 331.59 | 37.86 | 195.68 | 16.76 | 18.63 | 9.08 | 4.37 | 4.62 |
| Female | 59 | 430.29 | 102.57 | 142.69 | 320.5 | 41.32 | 183.56 | 18.82 | 20.53 | 10.29 | 5.63 | 3.87 |

**https://doi.org/10.5061/dryad.r7sqv9skk**
