# Supplementary material for: Evaluation of the horizontal approach to the medial malleolar facet in sagittal talar fractures through dorsiflexion and plantarflexion positions
Source: PLoS One. 2024 May 15;19(5):e0295350. doi: 10.1371/journal.pone.0295350 (PMC11095721; doi:10.1371/journal.pone.0295350)
Supplement: Attachment — Submitted filename: Response to Reviewers.doc [file pone.0295350.s002.doc]

*We deeply appreciate both the reviewers and the editors for their thoughtful comments and suggestions, which we have carefully read and studied. Please find below a detailed responses to your comments. And we do not know whether we focus on the point that you ask or not. If not, please give us some more information for this question. Thanks!*

| Numbered Reviewer Remark  and Manuscript Line Number | Author Response | Revised Manuscript Line  Number and Text Change |
| --- | --- | --- |
| 1.Please ensure that your manuscript meets PLOS ONE's style requirements, including those for file naming. | Revised |  |
| 2. We note that the grant information you provided in the ‘Funding Information’ and ‘Financial Disclosure’ sections do not match. | Revised |  |
| 3.Please remove any funding-related text from the manuscript and let us know how you would like to update your Funding Statement. | Removed from manuscript |  |
| 4. In your Data Availability statement, you have not specified where the minimal data set underlying the results described in your manuscript can be found. | Updated | DOI：10.5061/dryad.r7sqv9skk |
| 5.Please include your full ethics statement in the ‘Methods’ section of your manuscript file. In your statement, please include the full name of the IRB or ethics committee who approved or waived your study, as well as whether or not you obtained informed written or verbal consent. If consent was waived for your study, please include this information in your statement as well. | Revised | Line98-Line89 |
| 6.Please review your reference list to ensure that it is complete and correct. | No retractions were found. |  |
|  |  |  |
| **Reviewer 1:** |  |  |
| 33-35: change to “The talar model from Mimics was imported into Geomagic software for image refinement. Then Solidworks software was used to segment the medial surface of the talus and extend the 35 edge lines from the three positions to project them onto the "semicircular" base for 2D projection”. This can be edited for clarity as well. | Revised | Line 36-Line 38 |
| 39-50: Replace semicolons with periods (check for whole section for punctuation errors) | Revised | Line 43-Line 53 |
| 42: add “, respectively” | Revised | Line 46 |
| 42: remove “position” | Revised | Line 46 |
| 62: please standardize either “foot-and-ankle” or “foot and ankle” throughout the manuscript | Revised | Line 67 |
| 64: should be “falls | Revised | Line 69 |
| 68: please elaborate on “etc” or remove | Removed from manuscript | Line 73 |
| 68: add comma after reduced | Revised | Line 73 |
| 71: delete “surgeries in” | Revised | Line 76 |
| 78: The foot cannot be dorsiflexed and plantarflexed at the same time. Please clarify - replace “and” with “or” | Revised | Line 83 |
| 87: were patients retrospectively or prospectively recruited? Please clarify | Prospectively | Line 92 |
| 94: Was consent obtained for all patients? Or just the one whose radiographs are in figure 4? If all patients please updated the text. If only figure 4 please change the text to explain it that it is the one patient whose radiographs are displayed in the manuscript. | Obtained all patients. | Line 99-Line 100 |
| 95: what does “all methods were carried out in accordance with relevant guidelines”? Which methods and what guidelines? | This study was conducted in accordance with the World Medical Association Declaration of Helsinkia, removed “All methods were carried out in accordance with relevant guidelines”from manuscript | Line 97-Line 98 |
| 96: should be formatted “(range, 18-74 years)” please correct for the rest of the manuscript | Revised | Line 101-Line 102 |
| 108-111: make changes suggested in my comment for lines 33-35 | Revised | Line 114-Line 117 |
| 119: should be “in the functional position” correct if this error is found elsewhere in the manuscript | All revisions have been made in the manuscript. |  |
| 123: replace “measured the distances” with “the distances were measured” | Revised | Line 141-Line 143 |
| 124: write “the distance of the medial malleolar facet border …” … “(red line, L2), and the distance from the insertion point to the tuberosity of the medial malleolus facet …” | Revised | Line 141-Line 143 |
| 125: put the L1, L2, L3 as (L1) after the description. Ex: “from the insertion point to the top of the talar dome (L1), …” | Revised | Line 142-Line 144 |
| 125: I don’t think I’ve seen this wording before: tuberosity of medial malleolus facet of deltoid ligament posterior tibial talar. Please explain and re-word. Is this the posterior tibiotalar portion of the deltoid ligament complex? (Change this phrasing in wherever else it is applicable ex: Fig 3) | It is the posterior tibiotalar portion of the deltoid ligament complex. | Line 144 |
| 128: write out standard deviation instead of “SD” | Revised | Line 154 |
| 128: what kind of T test? Student t-test? Wilcoxin sign-rank? T tests were used to compare what data? | The independent sample T test. | Line 154 |
| 138, 141, 146: in what manner was the intersex difference significant? Please elaborate. Intersex difference in area? | Revised. | Line 163-Line 164;  Line 166-Line 167;  Line 178-Line 179 |
| 152-153: needs citation. Also, what are the differences between female and male? Also it should be “females and males” in the text | Reference have been added | Line 189 |
| 157-159: needs citation. What tendons, nerves, and vessels may be in the way. | Reference have been added | Line 194-Line 196 |
| 161: which factors? Please elaborate | Increased exposure, sufficient space, and joint locking | Line 198 |
| 162-164: need citations. which tendons? Which muscles? Provide study citation for muscle relaxation leading to greater exposure area. | Reference have been added | Line 200-Line 201 |
| 174-175: informed consent statement disrupts flow of discussion, remove (assuring that it can be found in the methods section) | Removed from manuscript | Line 212 |
| 181: replace obviously - too informal | Revised. | Line 218 |
| 182-183: As with 152-153, please describe the differences with literature. Also it should be “females and males” in the text | Revised. | Line 220 |
| 188-190: please cite literature on the reduced economic burden | No relevant literature was found.Removed “and economic burden”from manuscript | Line 231 |
| 193-194: please cite literature to support your claim that “Chinese people … have different skeletal shapes with American and European populations.” Also it should be different shapes “than” | References have been added | Line 235 |
| 195: plural of talus is tali, please update text | Revised. | Line 236 |
| 196-197: please elaborate on what is meant by “It is necessary to improve the quality of fracture reduction by preoperative 3D reconstruction and intraoperative use of reduction forceps.” How does this fit into the limitations section? | As mentioned earlier,we only analyzed the data based on genders, not different age, body size and type of fracture groups. These factors may also affect the visualization of the medial malleolus facet and the positioning of screws,and the study was performed on intact tali, but the normal anatomy is distorted in the case of talar fractures.Preoperative 3D reconstruction and intraoperative use of reduction forceps is a solution to these problems. In this study, 3D reconstruction is also used to evaluate the surgical approach. We think that it is reasonable to add this suggestion here. |  |
|  |  |  |
| **Reviewer 2:** |  |  |
| Line 46: This sentence requires correction for better clarity. Additionally, it would be beneficial to include information about UA in the abstract. | Revised. | Line 48-Line 50 |
| Consider organizing the Methods section into clearly defined subsections. This would greatly enhance the readability and logical flow of the paper. | Revised. | Line 92-Line 155 |
| Some content in the Methods section appears to pertain to the Results. Please review and relocate these sentences to the appropriate section. | Although "semicircular" region is the result of discovery, but we think that it is also the premise of the next operation. |  |
| For a more comprehensive analysis, consider using the proportion of each variable relative to the total talus surface area. This adjustment could account for variations in talus size due to factors like height and gender. | In this study, the difference between the values of FAA and FAP is not obvious, and the sample size is not large enough, and the evaluation error by height may be large and the evaluation is too far-fetched. We think that the area change caused by the change of body position is more clear and prominent by the difference value. |  |
| Lines 141-144 require rewriting for better clarity and coherence. | Revised. | Line 167-Line 169 |
