# Supplementary material for: Evaluation of the horizontal approach to the medial malleolar facet in sagittal talar fractures through dorsiflexion and plantarflexion positions
Source: PLoS One. 2024 May 15;19(5):e0295350. doi: 10.1371/journal.pone.0295350 (PMC11095721; doi:10.1371/journal.pone.0295350)
Supplement: Attachment — Submitted filename: Response to Reviewers.doc [file pone.0295350.s003.doc]

*We deeply appreciate both the reviewers and the editors for their thoughtful comments and suggestions, which we have carefully read and studied. Please find below a detailed responses to your comments. And we do not know whether we focus on the point that you ask or not. If not, please give us some more information for this question. Thanks!*

| Numbered Reviewer Remark  and Manuscript Line Number | Author Response | Revised Manuscript Line  Number and Text Change |
| --- | --- | --- |
| Please review your reference list to ensure that it is complete and correct. If you have cited papers that have been retracted, please include the rationale for doing so in the manuscript text, or remove these references and replace them with relevant current references. Any changes to the reference list should be mentioned in the rebuttal letter that accompanies your revised manuscript. If you need to cite a retracted article, indicate the article’s retracted status in the References list and also include a citation and full reference for the retraction notice. | No retracted papers. |  |
|  |  |  |
| **Reviewer 1:** |  |  |
| Whole article: There should be spaces before parenthesis “ (“ | Revised |  |
| 34:change to “In dorsiflexion, plantarflexion, and functional foot positions” and make sure this grammatical correction is applied to other instances. On line 110 “in the functional position, dorsiflexed, or plantarflexed” is also an acceptable way to phrase this. | Revised | Line 34;  Line 106-Line 107 |
| 36: delete extra period | Revised | Line 36 |
| 67: change all instances of “foot-and-ankle” to “foot and ankle” | Revised | Line 67;  Line 76;  Line 106 |
| 72: should be “and deformities” | Revised | Line 72 |
| 102: space before “years” | Revised | Line 102 |
| 169: remove and replace obviously with formal language | Removed from manuscript | Line 169 |
| Table 1: improve formatting for reader | Revised | Line 171-Line 173 |
| 189, Reference 14: this is a study on a pediatric population, however this study has a mean age of approx. 45, please find literature that represents the patients in your study. | Revised | Line 186 |
| 198: change comma after “locking” to ; or – | Revised | Line 195 |
| 199-203: run on sentence, please split into 2 sentences. “Additionally” should be the start of the second sentence. And please make sure that there are citations for the claims made in the first sentence. | Revised | Line 198-Line 200 |
| 219-220: same claim as 189. See above. | Revised | Line 217 |
| 234: change talus to tali | Removed | Line 231 |
| 235: be more specific than “skeletal shapes”. The shapes of which bones? What is the shape difference? What about the bone micro-architecture is different? Or remove sentence entirely. | Removed | Line 231 |
